# Catechol-Based Porous Organic Polymers for Effective Removal of Phenolic Pollutants from Water

**DOI:** 10.3390/polym15112565

**Published:** 2023-06-02

**Authors:** Xiaoxiao Zhao, Yiqiong Liu, Qimeng Zhu, Weitao Gong

**Affiliations:** School of Chemical Engineering, Dalian University of Technology, Dalian 116024, China; zxx1922@163.com (X.Z.); 18729470636@mail.dlut.edu.cn (Y.L.); zhuqimeng@mail.dlut.edu.cn (Q.Z.)

**Keywords:** catechol, biomass, porous organic polymers, adsorption, phenolic pollutants

## Abstract

Phenolic pollutants released from industrial activities seriously damage natural freshwater resources, and their elimination or reduction to safe levels is an urgent challenge. In this study, three catechol-based porous organic polymers, CCPOP, NTPOP, and MCPOP, were prepared using sustainable lignin biomass-derived monomers for the adsorption of phenolic contaminants in water. CCPOP, NTPOP, and MCPOP showed good adsorption performance for 2,4,6-trichlorophenol (TCP) with theoretical maximum adsorption capacities of 808.06 mg/g, 1195.30 mg/g, and 1076.85 mg/g, respectively. In addition, MCPOP maintained a stable adsorption performance after eight consecutive cycles. These results indicate that MCPOP is a potential material for the effective treatment of phenol pollutants in wastewater.

## 1. Introduction

Global population growth and industrial expansion have led to an increasing demand for freshwater resources, but water pollution has become a serious problem impeding freshwater supplies [1,2,3]. Phenolic pollutants in water present a particularly high potential risk because of their toxicity, non-biodegradability, and carcinogenicity, which makes them susceptible to human enrichment and poses serious health risks [4,5]. The development of new methods for the efficient removal of phenolic pollutants from water has become a pressing issue [6]. Adsorption [7,8] has the advantages of a wide processing range, low cost, and easy operation compared to other water treatment methods such as membrane separation [9,10,11], ion exchange [12,13,14,15], and electrochemical [16,17] and photodegradation [18,19]. However, conventional adsorbent materials such as activated carbon [20], chitosan [21], and mesoporous silica nanoparticles [22] have shown a relatively lower adsorption capacity and still need further improvement to meet the practical requirements for removing phenolic pollutants. In this sense, finding efficient, eco-friendly phenol adsorbents is always highly desired and represents a great challenge for relative researchers.

Porous organic polymers (POPs) are an emerging class of functionalized porous materials designed and assembled from organic precursors [23]. They have tremendous tunable functional properties in terms of high specific surface area, rich pore structure, and type/number of chemical groups [24,25,26,27]. However, most of the current POPs feedstock is derived from petrochemicals, and the development and reuse process inevitably results in secondary damage [28]. Accordingly, the development of sustainable renewable raw materials for the preparation of POPs as alternatives is attractive and essential. It is known that lignin is the most abundant natural renewable source of organic carbon as a supplementary resource to petroleum products [29]. The depolymerization of lignin provides various sustainable aromatic monomers [30,31], which can be used for the construction of novel biomass-derived POPs sorbents [32,33]. By selecting and assembling different biomass monomers and linkers, many fascinating POPs have been found to exhibit impressive adsorption capabilities [34,35,36,37]. Catechol has certain functional monomeric advantages as a typical lignin-derived monomer with hydrophilic o-hydroxy functional groups and different post-synthetic strategies [38]. Although some POPs prepared based on catechol have proved to have potential applications in the field of adsorption and separation, studies on their phenolic adsorption properties are still less explored [39,40].

Herein, to obtain green and efficient biomass adsorbents and to investigate the effect of catechol derivatives on the structure and adsorption properties of polymers, we chose sustainable catechol (Ccol), 2,3-naphthalene diol (Ntdiol), and 4-methyl catechol (Mcol) as monomers and formaldehyde dimethyl acetal (FDA) as a cross-linker to obtain three new POPs (CCPOP, NTPOP, and MCPOP), by using the Friedel–Crafts alkylation reaction. Cross-linked networks are formed by polymerization to form polymers with a high specific surface area, a rich pore structure, and a polyhydroxylated structure, thus enabling the more efficient adsorption of pollutants in water. The adsorption of three POPs for phenolic pollutants in water was studied systematically. As a result, three catechol-based POPs exhibit high phenol adsorption capacity compared with previously reported porous organic polymers. In particular, NTPOP and MCPOP reached 1195.30 mg/g and 1076.85 mg/g, which are greater than most POPs that have been reported. The difference between CCPOP, NTPOP, and MCPOP comes from the different structures of the catechol derivative monomers, which enable the three polymers to have different molecular sizes, specific surface areas, and phenolic pollutants’ adsorption capacities. The current work provides insights into the efficient adsorption of phenolic pollutants by sustainable biomass POPs.

## 2. Materials and Methods

### 2.1. Materials

Catechol (Ccol), 2,3-naphthalene diol (Ntdiol), and 4-methyl catechol (Mcol) were purchased from Shanghai McLean Biochemical Technology Co., Ltd. (Shanghai, China). Formaldehyde dimethyl acetal (FDA), bisphenol A (BPA), 4,4′-sulfonyldiphenol (BPS), phenol, 4-chlorophenol (4-CP), 2,4-dichlorophenol (DCP), and 2,4,6-trichlorophenol (TCP) were purchased from Sa’en Chemical Technology Co., Ltd. (Shanghai, China). 1,2-dichloroethane was purchased from Tianjin Fuyu Fine Chemical Co., Ltd. (Tianjin, China). FeCl_3_ was purchased from Tianjin Bodi Chemical Co., Ltd. (Tianjin, China). Methanol anhydrous was purchased from Tianjin Dongli District Tianda Chemical Reagent Factory (Tianjin, China). Activated carbon was purchased from Tianjin Beichen Fangzheng Reagent Factory (Tianjin, China).

### 2.2. Polymer Synthesis

**Scheme 1:**
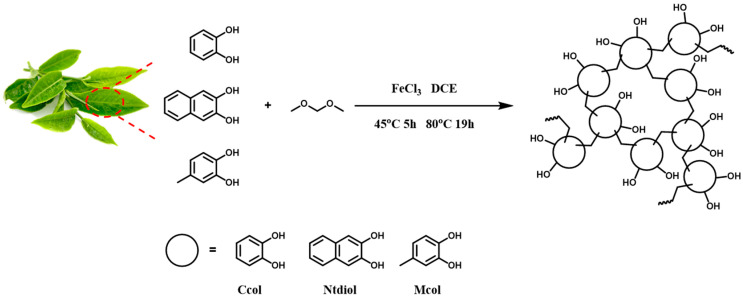
Chemical structures of catechol derivatives and the synthesis of POPs.

The polymer synthesis process is shown in Scheme 1, according to the previously reported work [38]. In a typical Schlenk tube, 20 mmol of Ccol, Mcol, or Ntdiol was dissolved in 30 mL of 1,2-dichloroethane solvent separately. Further, 40 mmol of FDA and FeCl_3_ was added to the reaction mixtures under dry conditions. The resulting mixtures were stirred for 5 h at 45 °C and then heated to 80 °C for 19 h. After cooling, the resulting solids were collected by filtration and washed with methanol solvent until colorless filtrates were found. Moreover, the reaction mixtures were purified with methanol by the Soxhlet extraction process for 24 h and dried under the vacuum pump to obtain polymers, i.e., CCPOP, MCPOP, and NTPOP, respectively.

### 2.3. Characterization

The Fourier transform infrared (FT-IR) spectra of the polymers were tested by the JASCO IR-4100 spectrometer (JASCO, Tokyo, Japan). The solid-state ^13^C cross-polarization with magic-angle spinning (CP/MAS) results were collected by a 599.7 MHz nuclear magnetic resonance spectrometer (JNM-ECZ600R, Agilent, Santa Clara, CA, USA). Powder X-ray diffraction (PXRD) results were obtained by using a Rigku D/max-2400 diffractometer (40 kV, 200 mA) from 5° to 80° with a scanning rate of 2°/min (Bruker AXS, Madison, WI, USA). The scanning electron microscopy (SEM) analysis was examined by using HITACHI-SU5000 (HITACHI, Tokyo, Japan). The results of the adsorption and desorption of N_2_ were obtained by an analyzer called Quantachrome-Autosorb IQ (Quntachrome, Kanagawa, Japan). The thermogravimetric (TGA) analysis was executed by using Mettler Toledo TGA/DSC 3+ (Mettler Toledo, Greifensee, Switzerland) under a nitrogen atmosphere. Samples were heated from 25–800 °C with a 10 °C/min heating rate. The ultraviolet (UV) spectra of the polymers were measured by the JASCO V-750 (JASCO, Tokyo, Japan). The X-ray photoelectron spectroscopy (XPS) analysis was examined by using ESCALAB XI+ (thermo, Oxford, UK).

### 2.4. Batch Adsorption Experiments

Phenol, 4-CP, DCP, TCP, BPA, and BPS were used as model pollutants to study the adsorption properties of the polymers (CCPOP, NTPOP, and MCPOP). Solutions of phenol contaminants with a certain concentration gradient were prepared, the absorbance at different concentrations was measured by a UV spectrophotometer, and the peak values at the corresponding wavelengths (λ_Phenol_ = 269.8 nm, λ_4-CP_ = 280 nm, λ_DCP_ = 284 nm, λ_TCP_ = 286.8 nm, λ_BPA_ = 276 nm, λ_BPS_ = 277 nm) were recorded. The standard curves were fitted to the absorbance concentrations according to the Lambert–Bier law. The actual concentration of the solution can be calculated from the standard curve. The polymeric adsorbents were added to an initial concentration of 100 mg/L of model aqueous solution (10 mL) for adsorption. After the adsorption experiment, the solid and liquid phases were separated by a 0.22 μm filter membrane syringe, and the filtrate was collected. The residual concentrations of phenol, 4-CP, DCP, TCP, BPA, and BPS in the filtrate were determined by UV spectrophotometry. The amount of adsorption at the equilibrium state ($Q_{e}$, mg/g) is calculated by the given equation.

(1) $$Q_{e}=\frac{(C_{0}-C_{e})V}{m}$$

in which $Q_{e}$ (mg/g) is the equilibrium adsorption capacity; $C_{0}$ and $C_{e}$ (mg/L) are the initial and final equilibrium concentrations of phenol, 4-CP, DCP, TCP, BPA, and BPS in solution; V (mL) is the volume of solution; and m (mg) is the mass of the adsorbent.

Adsorption kinetics experiments were carried out at room temperature by magnetic stirring. CCPOP, NTPOP, and MCPOP (30 mg) were added to TCP aqueous solution (150 mg/L, 80 mL) and BPA aqueous solution (50 mg/L, 60 mL), respectively. The concentrations of TCP and BPA were calculated at different times by UV spectrophotometry. Pseudo-first-order and pseudo-second-order kinetic models were used to analyze the kinetics of the adsorption of phenolic pollutants. The kinetic model equations for pseudo-first-order and pseudo-second-order are as follows.

(2) $$Q_{t}=Q_{e}\left(1-Q_{t}e^{-k_{1}t}\right)$$

(3) $$Q_{t}=\frac{k_{2}Q_{e}^{2}t}{1+Q_{e}k_{2}t}$$

in which $Q_{e}$ (mg/g) is the equilibrium adsorption amount, t (min) is the adsorption time, $Q_{t}$ (mg/g) is the phenolic pollutants adsorption amount at time t (min), $k_{1}$ (min^−1^) is the pseudo primary rate constant, and $k_{2}$ (g/(mg min)) is the pseudo secondary rate constant.

To assess the saturation adsorption capacity of the polymeric adsorbents for TCP and BPA, adsorption isotherms were tested at 25 °C. CCPOP, NTPOP, and MCPOP (4 mg) were added to an aqueous solution of TCP (concentration: 100–500 mg/L, 10 mL); CCPOP, NTPOP, and MCPOP (5 mg) were added to an aqueous solution of BPA (concentration: 50–300 mg/L, 10 mL) and adsorbed while stirring to ensure saturation of adsorption. The Langmuir and Freundlich models were used to quantify and compare the adsorption performance of different polymeric adsorbents (CCPOP, NTPOP, and MCPOP). The equations for the Langmuir and Freundlich models are as follows.

(4) $$Q_{e}=\frac{Q_{m}K_{L}C_{e}}{1+K_{L}C_{e}}$$

(5) $$lnQ_{e}=lnK_{F}+\frac{1}{n}lnC_{e}$$

in which $K_{L}$ and $K_{F}$ are the constants of the Langmuir and Freundlich models, respectively, $\frac{1}{n}$ is the empirical parameter of the Freundlich model, $C_{e}$ (mg/g) is the equilibrium concentration of phenolic pollutants, $Q_{e}$ (mg/g) is the equilibrium adsorption capacity, and $Q_{m}$ (mg/g) is the maximum adsorption capacity.

### 2.5. Adsorption Cycling Experiments

The adsorbent is desorbed and regenerated by immersion in a solution of acetone and ethanol. The precipitate was collected by centrifugal precipitation and dried at 80 °C for the next cycle. The regeneration performance of the adsorbent was explored through eight consecutive adsorption–desorption cycles. The pollutant removal efficiency after each cycle was calculated separately. Equilibrium removal efficiency (%) is calculated using the following equations.

(6) $$Removal\;efficiency\;(\%)=\left(\frac{C_{0}-C_{e}}{C_{0}}\right)\times 100\%$$

where $C_{0}$ and $C_{e}$ (mg/L) are the initial and final equilibrium concentrations of TCP in solution. The initial TCP concentration was 200 mg/L.

### 2.6. Adsorption Mechanism

To understand the mechanism of interaction between MCPOP and TCP, pH experiments and FT-IR spectroscopy were carried out. In the pH effect experiments, MCPOP (5 mg) was added to TCP aqueous solution (200 mg/L, 10 mL) at different pH (pH adjusted by HCl and NaOH), and the residual concentration was measured to calculate the adsorption amount.

## 3. Results and Discussion

### 3.1. Characterization of the Adsorbents

**Figure 1:**
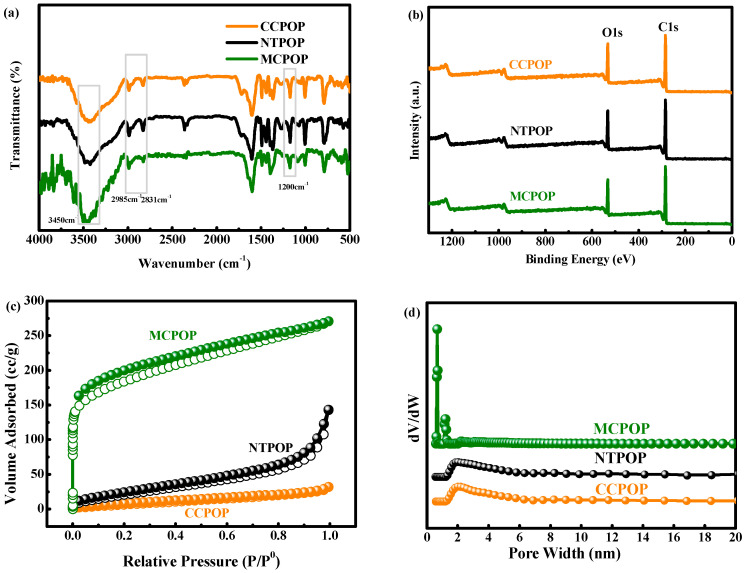
(**a**) Fourier transform infrared (FT-IR); (**b**) X-ray photoelectron spectroscopy (XPS); (**c**) N_2_ adsorption–desorption curves; (**d**) pore-size distribution curve of CCPOP, NTPOP, and MCPOP.

The FT-IR spectra of CCPOP, NTPOP, and MCPOP are shown in Figure 1a. For CCPOP, NTPOP, and MCPOP, the peaks at 2985 cm^−1^ and 2831 cm^−1^ (stretching vibration of C−H) are ascribed to the methylene group of the FDA. The source of the bands at 3500 cm^−1^ and 1200 cm^−1^ (O−H and C−O) is the hydroxyl group fixed on the aromatic ring. In addition, three polymers were further characterized by solid-state ^13^C cross-polarization magic-angle spinning (CP-MAS) NMR spectroscopy. As shown in Figure S1, the high-intensity characteristic resonance observed at 14–50 ppm represents the methylene signal of cross-linking with FDA in the framework, which indicates that the catechol-derived monomers were successfully cross-linked with FDA. Furthermore, the characteristic resonances observed at around 120–150 ppm represent the aromatic and non-substituted aromatic carbons on phenolic hydroxyl groups, and the specific resonance peaks indicate that the polymer is a chemical structure cross-linked by catechol monomers.

**Figure 2:**
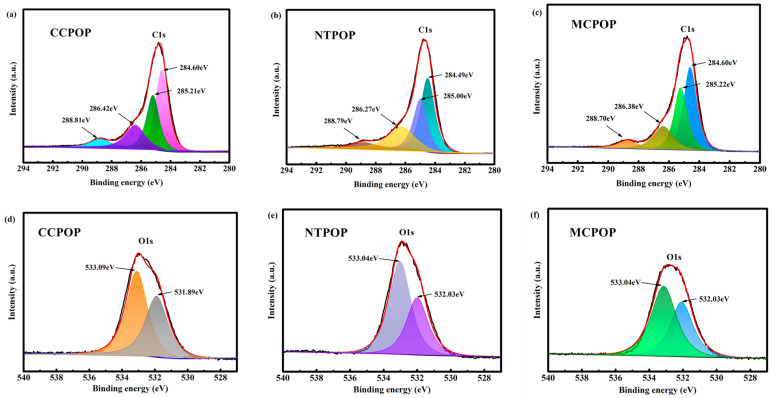
C1s (**a**) and O1s (**d**) spectra of CCPOP, C1s (**b**) and O1s (**e**) spectra of NTPOP, and C1s (**c**) and O1s (**f**) spectra of MCPOP.

Powder X-ray diffraction (PXRD) plots show a broad rather than a sharp peak at around 2θ = 20° (Figure S2), indicating that all three polymers are not crystalline but amorphous structures. The XPS measurements were performed to investigate the elemental composition of CCPOP, NTPOP, and MCPOP. The XPS images (Figure 1b) indicated the existence of C and O in the CCPOP, NTPOP, and MCPOP. A consecutive series of binding energies in the range of 284.4–284.6 eV, 285.0–285.22 eV, 286.27–286.42 eV, and 288.70–288.81 eV were distinguished in the C1s XPS spectra (Figure 2a–c), which could be attributed to C=C, C−C, C−OH and C=O functional groups, respectively. The C−OH and C=O photoelectron peaks approximately appeared at 533.05 and 532.00 eV, respectively. In addition, the surface morphology of the polymers was investigated by scanning electron microscopy (SEM). As shown in Figure S3, after cross-linking to form the polymers, CCPOP and NTPOP are composed of fused small particles with partially spherical structures; in contrast, MCPOP shows a porous structure and irregular surface state. FeCl_3_ is loaded into the polymer by complexation with the O atoms in the catechol monomer [41].

The permanent porosities and surface areas of the three polymers were further measured and calculated by using N_2_ adsorption–desorption isotherm at 77 K. As shown in Figure 1c, the specific surface areas of CCPOP, NTPOP, and MCPOP were 33.89 m^2^/g, 93.26 m^2^/g, and 665.97 m^2^/g, respectively. MCPOP formed a type I adsorption and desorption curve while CCPOP and NTPOP formed type III adsorption and desorption graphs. Combined with pore-size distribution (PSD) (Figure 1d), the significant increase in adsorption at low pressure indicates a large number of micropores in the MCPOP. As shown in Table 1, the pore volume of MCPOP is significantly larger compared to CCPOP and NTPOP, indicating a better degree of cross-linking of MCPOP. MCPOP had large S_BET_ and abundant pore space, which can be attributed to two reasons: (1) Different catechol monomers have different effects on the pore size, pore volume, and specific surface area of the polymer. Mcol as a reaction precursor significantly increases the pore volume and specific surface area of the polymer. The spatial site resistance effect is also an important factor. (2) The adjacent hydroxyl structure may not be conducive to Friedel-Crafts reaction cross-linking to form a network structure, as the FeCl_3_ catalyst tends to complex with the O atoms to deactivate the catalyst [31].

TGA was used for the thermal analysis of the three catechol polymers, as shown in Figure S4. First, the weight loss between room temperature and 100 °C was caused by the evaporation of water from the samples. The weights of the three catechol polymers remained essentially constant between 100 °C and 250 °C, indicating that the evaporation of crystalline water from the polymers was complete. Between 250 °C and 450 °C, the polymer weight loss increased sharply, presumably due to the decomposition of the polymers.

### 3.2. Batch Adsorption Experiments

**Figure 3:**
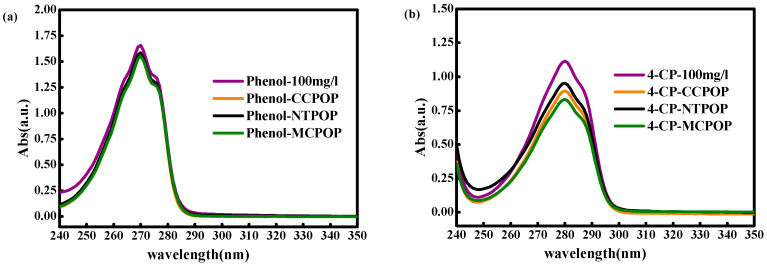

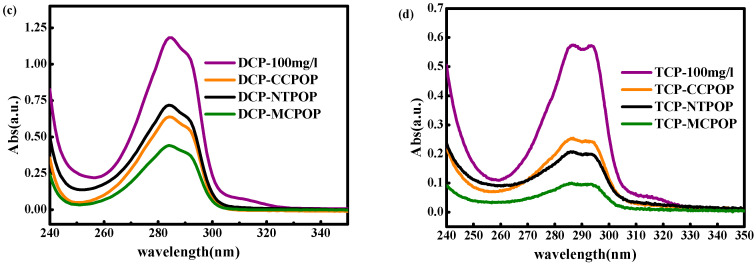
UV-vis spectra of (**a**) phenol, (**b**) 4-CP, (**c**) DCP, and (**d**) TCP aqueous solution. (Adsorbents: 5 mg, initial concentration: 100 mg/L, t = 8 h).

To investigate the adsorption performance of the three polymers on phenolic pollutants in water, batch adsorption experiments were first performed on phenol, 4−CP, DCP, TCP, BPA, and BPS, as shown in Figure 3 and Figure S5. The 100 mg/L TCP solution was passed through the filter membrane as a blank control experiment. The error of approximately 4% is within reasonable limits. When the initial concentration of phenolic pollutants in water was 100 mg/L, all three polymers were able to adsorb them to some extent, and the adsorption amounts are shown in Table S1. Comparing the three polymer adsorbents, MCPOP had the highest adsorption capacity. For chlorophenols, the adsorption capacity of the adsorbent increased with the number of −Cl substituents on the benzene ring. Chlorophenol adsorption mainly relies on hydrogen bonding, and the more the number of −Cl substituents increases, the easier it is to form hydrogen bonds between the adsorbent and the chlorophenols. This leads to the significant adsorption of TCP by the polymer. In addition, CCPOP, NTPOP, and MCPOP also showed high adsorption amounts for BPA (0.3536 mmol/g, 0.2712 mmol/g, and 0.6368 mmol/g). Therefore, TCP and BPA were selected to further investigate the adsorption properties of the polymers.

### 3.3. Adsorption Kinetics

**Figure 4:**
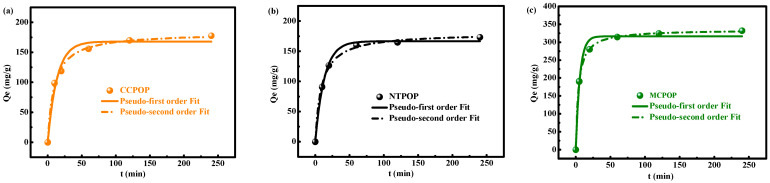
Kinetic modeling of TCP adsorption onto (**a**) CCPOP, (**b**) NTPOP, and (**c**) MCPOP. (Adsorbents: 30 mg, initial concentration: 150 mg/L, V = 80 mL, temperature: 25 °C).

To study the adsorption capacity of polymeric adsorbents on TCP and BPA, we investigated the adsorption kinetics of TCP and BPA. The data are shown in Figure 4a–c and Figure S6a–c.

The adsorption of TCP rapidly increased in the initial stage of adsorption, indicating that CCPOP, NTPOP, and MCPOP have significant adsorption affinity for TCP. Moreover, during the adsorption of BPA, MCPOP reached the adsorption equilibrium within 60 min, and CCPOP and MCPOP reached the adsorption equilibrium within 480 min. The adsorption efficiency of the adsorbents on TCP and BPA was evaluated by pseudo-first-order and pseudo-second-order kinetic models. The corresponding parameters and correlation coefficients obtained from the two models are listed in Table 2 and Table S2. Predictably, for CCPOP, NTPOP, and MCPOP adsorbents, pseudo-second-order kinetics can better describe TCP and BPA adsorption (R^2^ > 0.996), indicating that the adsorption of CCPOP, NTPOP, and MCPOP is mainly by chemisorption.

### 3.4. Adsorption Isotherms

The maximum adsorption capacities of polymeric adsorbents for TCP and BPA were evaluated by adsorption isotherms.

**Figure 5:**
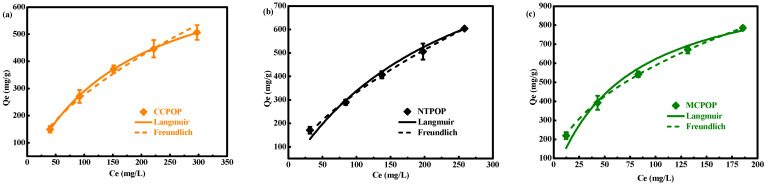
Isothermal adsorption curves of TCP onto (**a**) CCPOP, (**b**) NTPOP, and (**c**) MCPOP. (Adsorbents: 4 mg, V = 10 mL, temperature: 25 °C).

The initial concentrations of aqueous solutions of TCP were in the range of 100–500 mg/L, and the initial concentrations of BPA were in the range of 50–300 mg/L. The adsorption processes of TCP and BPA were fitted using Langmuir and Flanders models, as shown in Figure 5a–c and Figure S7a–c, and the relevant parameters and correlation coefficients are listed in Table 3 and Table S3. The maximum adsorption amounts of TCP by CCPOP, NTPOP, and MCPOP were 808.06 mg/g, 1195.30 mg/g, and 1076.85 mg/g, respectively; the maximum adsorption amounts of BPA by CCPOP, NTPOP, and MCPOP were 203.94 mg/g, 272.94 mg/g, and 264.48 mg/g, respectively. The adsorption capacities of several representative materials in phenol solutions are presented in Table 4. The results illustrated that NTPOP and MCPOP have a higher adsorption capacity in solution compared to the reported adsorbent materials [42,43,44,45,46,47]. The adsorption capacity of activated carbon for TCP is 16.23 mg/g. The new polymers have a higher adsorption capacity for TCP. The higher saturation adsorption capacity can be attributed to the large specific surface area of the polymeric adsorbent, the rich pore structure, and the presence of a large number of o-hydroxyl groups on the benzene ring of the polymer. The adsorption process is influenced by several different factors such as hydrogen bonding, mass transfer, and π−π interactions. The polymers are structurally different, but the combined effect of many factors results in similar adsorption capacities.

### 3.5. Adsorption Cycling

**Figure 6:**
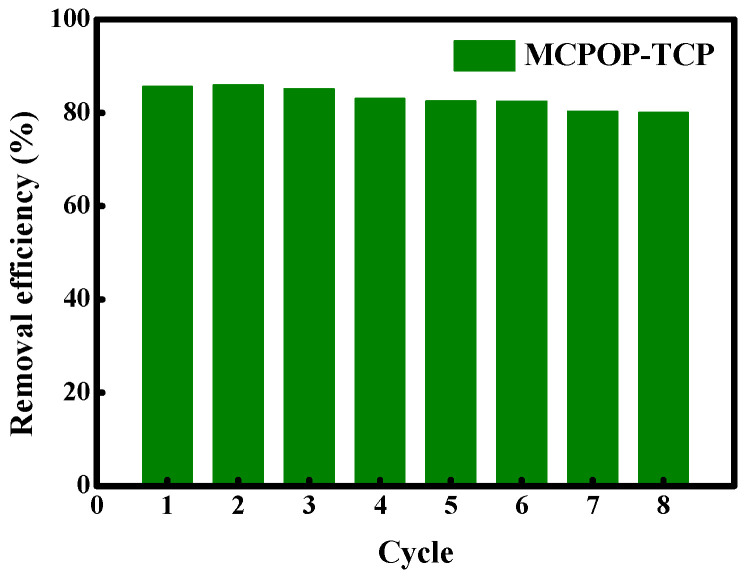
Removal efficiency of TCP by MCPOP in different cycles. (Adsorbents: 5 mg, V = 10 mL, C_0_ = 200 mg/L, t = 600 min).

The recoverability of phenolic adsorbents is an important indicator for achieving industrial applications. Specifically, the recoverability of MCPOP for TCP was investigated by cyclic adsorption–desorption experiments. As shown in Figure 6, after eight adsorption–desorption regeneration cycles, the removal efficiency of TCP decreased slightly and remained above 80%; presumably, the decrease in removal was mainly due to the partial loss of polymeric adsorbent during the cyclic regeneration process.

### 3.6. Adsorption Mechanism

To further analyze the adsorption mechanism of MCPOP-adsorbed TCP, we performed pH experiments and FT-IR spectroscopy studies on MCPOP.

**Figure 7:**
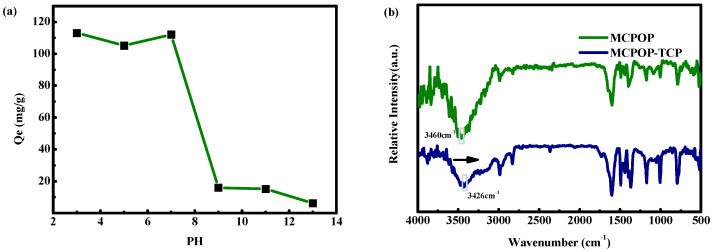
(**a**) The effect of pH on the adsorption of TCP by MCPOP; (**b**) FT-IR spectra of before and after loading phenolic pollutants.

As shown in Figure 7a, the adsorption of MCPOP was high and relatively stable when the pH was in the range of 3.0 to 8.0, but it decreased sharply as the pH in the solution continued to increase. It is presumed that this is due to the protonation of TCP under acidic conditions, which gives MCPOP the characteristics of strong electrostatic attraction and adhesion to TCP. However, under alkaline conditions, TCP deprotonates and forms electrostatic repulsion, resulting in the poorer adhesion of ionized MCPOP.

**Figure 8:**
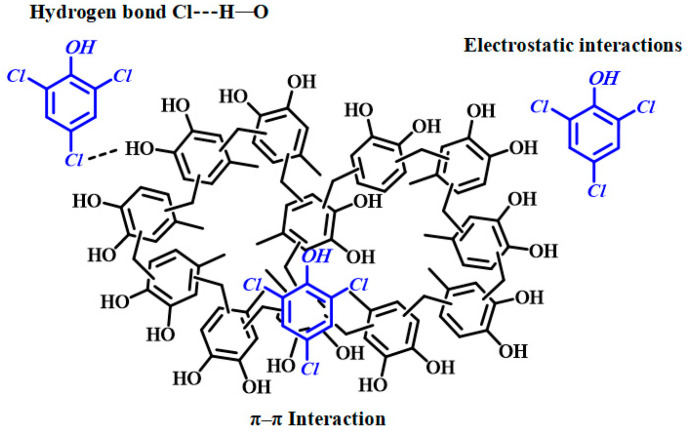
Adsorption mechanism of MCPOP for TCP.

The FT-IR spectra of MCPOP polymer adsorbent before and after the adsorption of TCP are shown in Figure 7b, where the original −OH of MCPOP was red-shifted (from 3372 to 3341 cm^−1^) after adsorption, indicating the formation of hydrogen bonds between MCPOP and TCP [48]. Based on the above adsorption model fitting, pH experiments, and instrumental analysis results, Figure 8 shows a schematic representation of the adsorption of TCP by MCPOP, elucidating the electrostatic interactions, π–π interactions, and hydrogen bonding interactions between the adsorbent and the contaminant.

## 4. Conclusions

In this study, three new polyhydroxy biomass POPs, CCPOP, NTPOP, and MCPOP, were successfully constructed by cross-linking for sustainable catechol as the functional monomer and FDA as the linker. The small structural variations of the catechol derivatives produced significant differences in the specific surface area of the polymers, with CCPOP having a specific surface area of 33.89 m^2^/g and MCPOP having a specific surface area of 665.97 m^2^/g. As a result of the adsorption, the POPs showed highly efficient phenolic pollutants adsorption capacities of 808.06 mg/g (CCPOP), 1195.30 mg/g (NTPOP), and 1076.85 mg/g (MCPOP), which is much better than previously reported for POPs adsorbent materials, especially biomass materials. Moreover, the MCPOP in cycling experiments maintained an adsorption efficiency of over 80% after 10 cycles, which is more suitable for complex and realistic environments. This study is expected to facilitate the application of bio-based porous polymers in the field of phenolic pollutant adsorption.

## Figures and Tables

**Table 1:** Information on the polymers’ specific surface area, pore volume, and main pore size.

Samples	S_BET_ (m^2^/g)	V_Total_ (cm^3^/g)	Pore Size (nm)
CCPOP	33.89	0.05	2.38
NTPOP	93.26	0.22	2.00
MCPOP	665.97	0.42	0.67

**Table 2:** Parameters of the pseudo-first-order and pseudo-second-order models of adsorption TCP.

Models	Parameters	CCPOP	NTPOP	MCPOP
Pseudo-first-order model	Q_e_ (mg/g)	167.9811	166.6401	316.5042
	k_1_ (min^−1^)	0.0732	0.0746	0.1718
	R^2^	0.9751	0.9950	0.9840
Pseudo-second-order model	Q_e_ (mg/g)	182.6841	180.6428	335.2129
	k_2_ (g·mg^−1^·min^−1^)	5.7793 × 10^−4^	6.0143 × 10^−4^	7.7736 × 10^−4^
	R_2_	0.9967	0.9978	0.9999

**Table 3:** Parameters of Langmuir and Freundlich adsorption isotherm models of adsorption TCP.

Models	Parameters	CCPOP	NTPOP	MCPOP
Langmuir	Q_m_ (mg/g)	808.06	1195.30	1076.85
	K_L_ (L/mg)	0.0056	0.0039	0.0138
	R^2^	0.9998	0.9927	0.9664
Freundlich	*n*	0.6027	1.6035	0.4672
	K_F_ [(mg·g^−1^)(mg·L^−1^)^−1/n^]	17.2516	17.9525	68.5325
	R^2^	0.9838	0.9991	0.9999

**Table 4:** Comparison of adsorption rates and $Q_{m}$ with reported adsorbents.

Absorbents	Equilibrium Time (min)	Q_m_ (mg/g)	References
[Zn(hba)_2_(tmdp)]_n_	180	207.8	[42]
GO-PVPP	240	466.7	[43]
TEPM-MON	3	294.6	[46]
β-CDP	10	108.0	[47]
PCD-PCP(L)	150	816.5	[48]
PCD-PCP(H)	150	647.6	[48]
CCPOP	120	808.06	This work
NTPOP	120	1195.30	This work
MCPOP	60	1076.85	This work

## Data Availability

All data related to this study are presented in this publication.

## Supplementary Materials

The following supporting information can be downloaded at: https://www.mdpi.com/article/10.3390/polym15112565/s1, Figure S1: Solid-state ^13^C NMR spectra for CCPOP, NTPOP, and MCPOP; Figure S2: X-ray diffraction (XRD) of CCPOP, NTPOP, and MCPOP; Figure S3: SEM images for CCPOP, NTPOP, and MCPOP; Figure S4: TGA curves for CCPOP, NTPOP, and MCPOP; Figure S5: UV-vis spectra of BPA and BPS aqueous solution; Table S1: The adsorption capacity of phenol, 4-CP, DCP, TCP, BPA, and BPS for CCPOP, NTPOP, and MCPOP. (Initial solution concentration: 100 mg/L); Figure S6: Kinetic modeling of BPA adsorption onto CCPOP (a), NTPOP (b), and MCPOP (c). (Adsorbents: 30 mg, initial concentration: 50 mg/L, V = 60 mL, temperature: 25 °C); Table S2: Parameters of the pseudo-first-order and pseudo-second-order models of adsorption BPA; Figure S7: Isothermal adsorption curves of BPA onto CCPOP (a), NTPOP (b), and MCPOP (c). (Adsorbents: 5 mg, V = 10 mL, temperature: 25 °C); Table S3: Parameters of Langmuir and Freundlich adsorption isotherm models of adsorption BPA.
