# Supplementary material for: Catechol-Based Porous Organic Polymers for Effective Removal of Phenolic Pollutants from Water
Source: Polymers (Basel). 2023 Jun 2;15(11):2565. doi: 10.3390/polym15112565 (PMC10255832; doi:10.3390/polym15112565)
Supplement: Supplementary file 1 [file polymers-15-02565-s001.zip › polymers-2376592-supplementary.pdf]

polymers

Supporting Information

Catechol-Based Porous Organic Polymers for Effective Removal of Phenolic Pollutants from Water

Xiaoxiao Zhao , Yiqiong Liu , Qimeng Zhu, and Weitao Gong *

School of Chemical Engineering, Dalian University of Technology, Dalian 116024, China; zxx1922@163.com (X.Z.); 18729470636@mail.dlut.edu.cn (Y.L.); zhuqimeng@mail.dlut.edu.cn (Q.Z.)

* Correspondence: wtgong@dlut.edu.cn; Tel.: +159-0426-8498

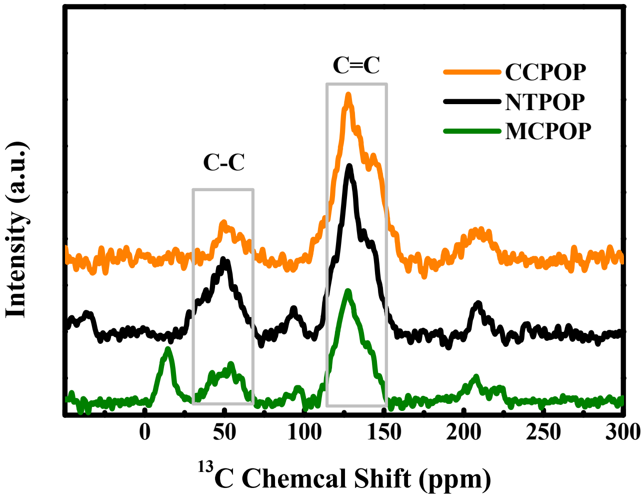

Figure S1. Solid-state ^{13}C NMR spectra for CCPOP, NTPOP and MCPOP.

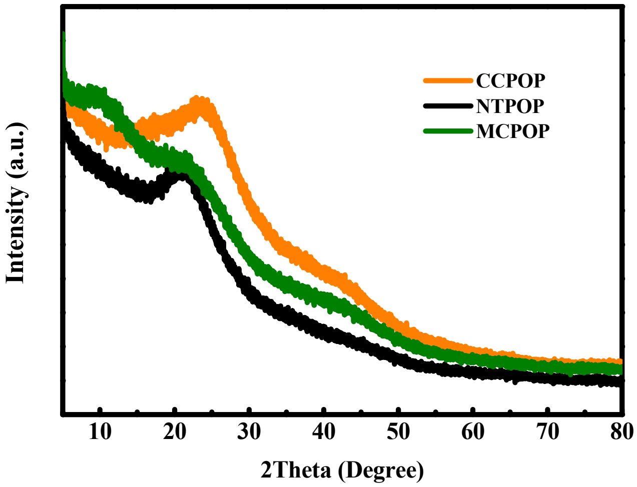

Figure S2. X-ray diffraction (XRD) of CCPOP, NTPOP and MCPOP.

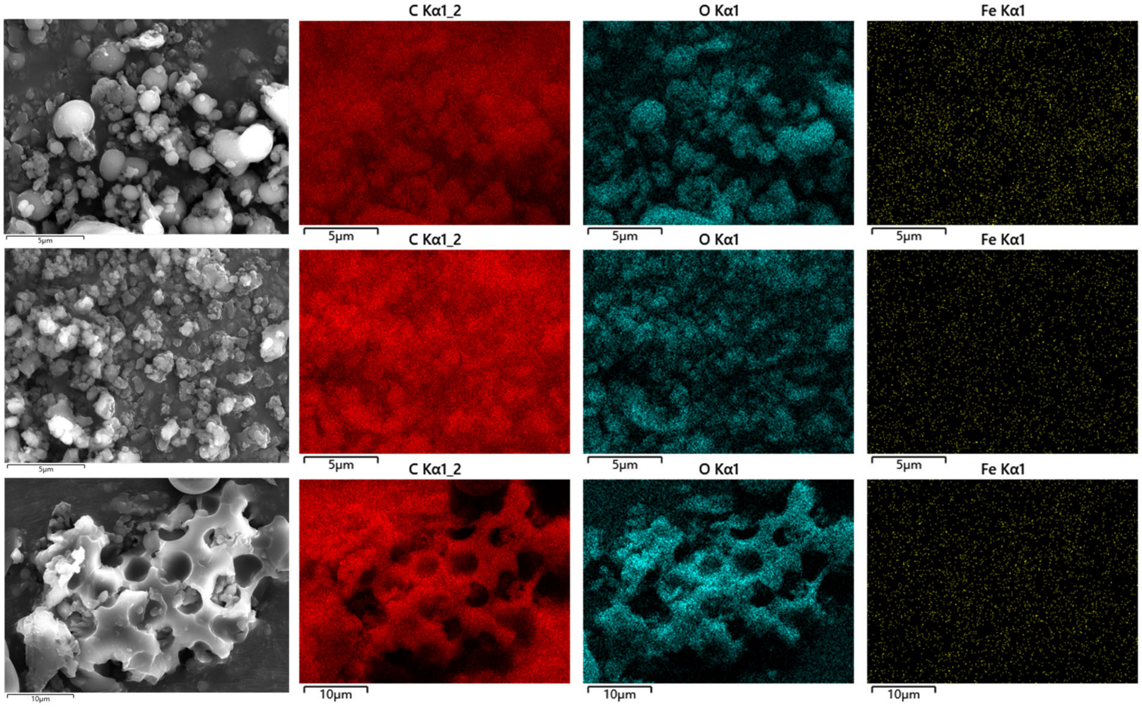

Figure S3. SEM images for CCPOP, NTPOP and MCPOP.

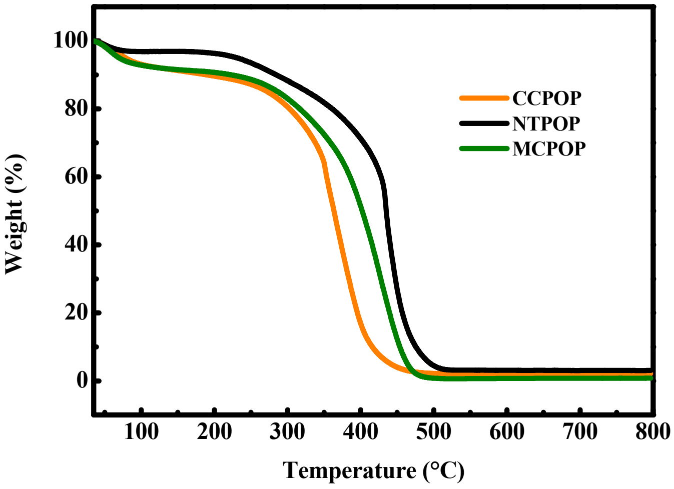

Figure S4. TGA curves for CCPOP, NTPOP and MCPOP.

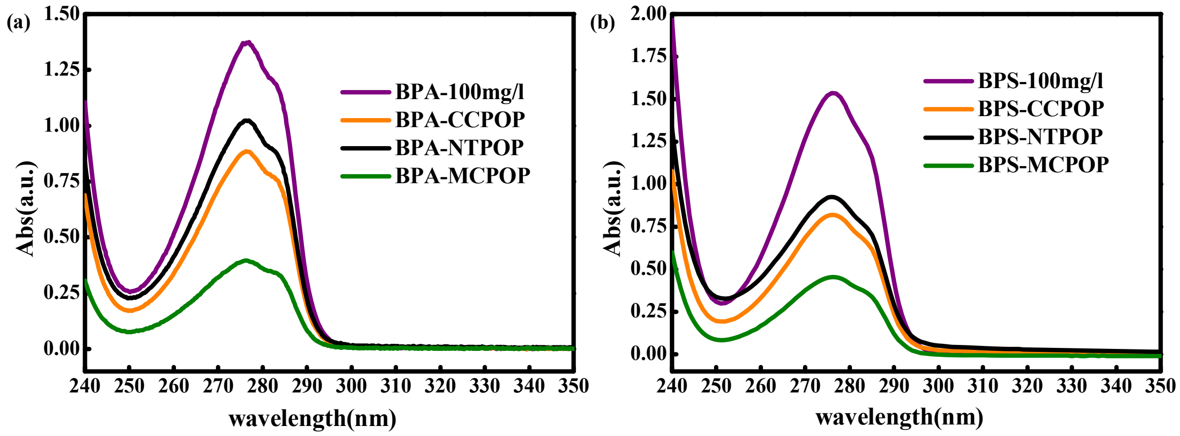

Figure S5. UV-vis spectra of BPA and BPS aqueous solution. (Adsorbents: 5 mg, initial concentration: 100 mg/L, t=8 h)

Table S1. The adsorption capacity of Phenol, 4-CP, DCP, TCP, BPA and BPS for CCPOP, NTPOP, and MCPOP. (Adsorbents: 5 mg, initial concentration: 100 mg/L, t=8 h)

Qe (mmol/g)	CCPOP	NTPOP	MCPOP
Phenol	0.2977	0.2429	0.3547
4-CP	0.3034	0.2214	0.3929
DCP	0.5724	0.4860	0.6590
TCP	0.6061	0.6920	0.8952
BPA	0.3536	0.2712	0.6368
BPS	0.3783	0.3205	0.5818

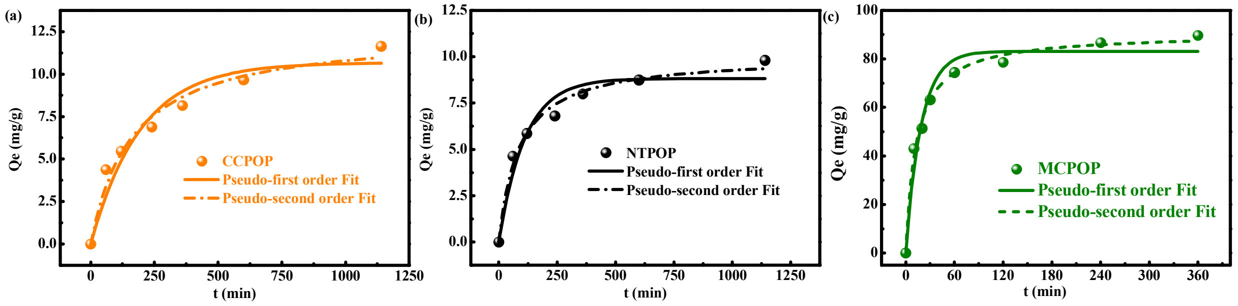

Figure S6. Kinetic modeling of BPA adsorption onto (**a**) CCPOP , (**b**) NTPOP and (**c**) MCPOP. (Adsorbents: 30mg, initial concentration: 50 mg/L, V=60mL, temperature: 25 °C)

Table S2. Parameters of the pseudo-first-order and pseudo-second-order models of adsorption BPA.

Models	Parameters	CCPOP	NTPOP	MCPOP
	Q_e (mg·g^{-1})	11.8011	9.5069	83.1458
**Pseudo-first-order model**	k_1 (min^{-1})	0.0040	0.0072	0.0528
	R^2	0.9348	0.9367	0.9620
	Q_e (mg·g^{-1})	13.1577	10.3661	90.5087
**Pseudo-second-order model**	k_2 (g·mg^{-1}·min^{-1})	4.1122×10^{-4}	10.2×10^{-4}	8.4562×10^{-4}
	R^2	0.97699	0.9868	0.9922

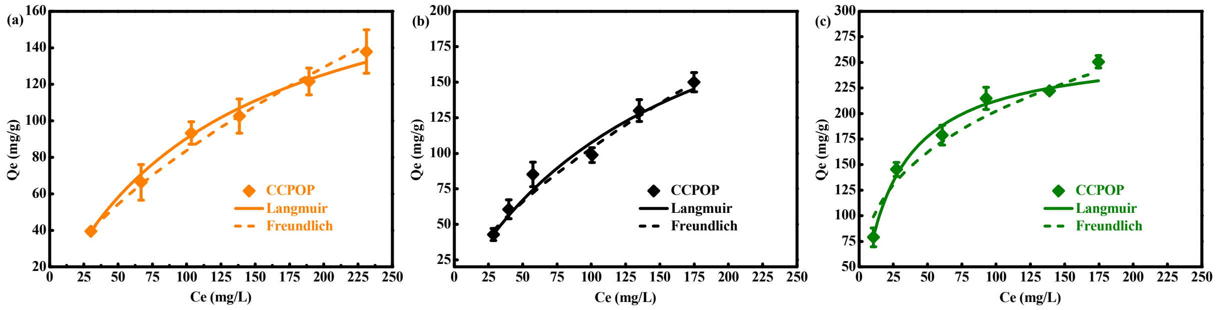

Figure S7. Isothermal adsorption curves of BPA onto (**a**) CCPOP, (**b**) NTPOP and (**c**) MCPOP. (Adsorbents: 5mg, V=10mL, temperature: 25 °C)

Table S3. Parameters of Langmuir and Freundlich adsorption isotherm models of adsorption BPA.

**Models**	**Parameters**	**CCPOP**	**NTPOP**	**MCPOP**
	Q_m (mg/g)	203.94	272.94	264.48
**Langmuir**	K_L (L/mg)	0.0080	0.0065	0.0407
	R^2	0.9971	0.9702	0.9557
	n	0.6251	0.6560	0.3179
**Freundlich**	K_F $[(mg \cdot g^{-1})(mg \cdot L^{-1})^{-1/n}]$	4.7110	5.0533	46.7732
	R^2	0.9924	0.9775	0.9537
